# Determinants of Adherence with Antimicrobial Resistant Organism (ARO) Admission Screening in a Provincial Health Care System

**DOI:** 10.1017/ash.2025.210

**Published:** 2025-09-24

**Authors:** Jenine Leal, Janice Pitchko, Bonita Lee, Jennifer Parsonage, Philana Choo, Logan Armstrong, John Conly, Jared Dembicki, Jennifer Ellison, Jayna Holroyd-Leduc, Sara Mallinson, Janine Phipps, Colin Reynolds, Geraldine St Jean, Joseph Vayalumkal, Katelyn Wiley, Samantha Woolsey, Elissa Rennert-May, Khara Sauro

**Affiliations:** 1 Alberta Health Services/University of Calgary; 2 Alberta Health Services; 3 University of Alberta; 4 Infection Prevention and Control, Covenant Health; 5 Foothills Medical Centre; 6 Infection Prevention & Control, Alberta Health Services; 7 University of Calgary; 8 Alberta Childrens Hospital

## Abstract

**Background:** Effective integration of antimicrobial resistant organism (ARO) admission screening into clinical information systems (CIS) can facilitate prompt identification of patients at risk of an ARO and interrupt transmission. However, ARO admission screening remains suboptimal in Alberta, Canada following implementation of the ARO admission screening tool in the provincial CIS. We sought to understand the determinants of adherence with the use of the ARO admission screening tool in the CIS. **Methods:** A mixed-methods study was conducted using a survey, human factors observations, and qualitative focus groups. Eligible participants included nursing staff and physicians from emergency departments and inpatient units in acute care and acute rehabilitation facilities where the ARO admission screening tool was utilized in the CIS in Alberta, Canada from September 6, 2023 to June 18, 2024 (n=100). A survey (REDCap) explored staff perceptions and experiences using the tool in the CIS. Observations and interviews of nursing staff completing the tool were guided by the Systems Engineering Initiative for Patient Safety model. Virtual (Zoom) semi-structured focus groups explored barriers and enablers of using the tool guided by the Theoretical Domains Framework. Descriptive analysis of survey responses was conducted using Microsoft Excel (Version 2409). Field notes and focus group transcripts were used for a rapid qualitative, thematic analysis. A weaving narrative by theme was used to integrate survey results with findings from the observations and focus groups. **Results:** There were 527 survey respondents representing all 5 health zones, 5 nurses observed and 20 interviews conducted by the human factors team, and 24 participants in 6 focus groups. Focus group participants represented different sized hospitals (12-1,099 beds) with varying ARO admission adherence rates (29-83%). Three emergent themes arose: context, the ARO admission screening tool, and the individual. Contextual factors included time constraints, increasing nursing workload, competing priorities, lack of patient cooperation, and a need to increase interactions with infection prevention and control programs. Attributes of the tool impacting completion included location of the tool within the CIS, lack of prompts, and multiple sources of information required to complete the tool. At an individual level, themes arose related to experience, perceptions of ARO screening, and lack of training that influenced completion of the tool. **Conclusions:** Among the emergent themes, multiple determinants were identified influencing the use of the ARO admission screening tool in the provincial CIS. These findings will help inform future strategies to improve ARO admission screening and reduce ARO transmission.

